# Preferential Activity of *Petiveria alliacea* Extract on Primary Myeloid Leukemic Blast

**DOI:** 10.1155/2020/4736206

**Published:** 2020-12-11

**Authors:** Ricardo Ballesteros-Ramírez, Eliana Aldana, María Victoria Herrera, Claudia Urueña, Laura Yinneth Rojas, Luis Fernando Echeverri, Geison Modesti Costa, Sandra Quijano, Susana Fiorentino

**Affiliations:** ^1^Grupo de Inmunobiología y Biología Celular, Unidad de Investigación en Ciencias Biomédicas, Facultad de Ciencias, Pontificia Universidad Javeriana, Bogotá, Colombia; ^2^Servicio de Hematología, Hospital Universitario San Ignacio, Bogotá, Colombia; ^3^Grupo de Química Orgánica de Productos Naturales (QOPN), Facultad de Ciencias Exactas y Naturales, Universidad de Antioquia, Medellín, Colombia; ^4^Grupo de Investigación Fitoquímica Universidad Javeriana, Departamento de Química, Facultad de Ciencias, Pontificia Universidad Javeriana, Bogotá, Colombia

## Abstract

The need for new therapeutic approaches to improve the response in acute leukemia (AL), either by directing therapy or with new therapeutic alternatives, has been a research and clinical interest topic. We evaluated whether blasts from AL patients were sensitive ex vivo to the induction chemotherapy and whether the extracts of *Petiveria alliacea* (Anamu SC) and *Caesalpinia spinosa* (P2Et) modulated the sensitivity of leukemic cells to death. Bone marrow samples were taken from 26 patients with de novo AL and 6 in relapse, and the cytotoxicity of the extracts alone or in combination with the chemotherapeutic was evaluated by XTT. Patients were classified as good (GR) and bad responders (BR) according to the ex vivo test. 70.5% of the GR patients to the ex vivo test achieved postinduction remission to induction chemotherapy with a median overall survival of 12.50 months versus 7.23 months in the two groups. Furthermore, it was found that the ex vivo response to extracts and chemotherapeutics is heterogeneous and shows an exclusive pattern between the extracts, Anamu being the more effective in inducing cell death. The combination of extracts with chemotherapeutic agents showed synergistic or antagonistic effects in the patients' blasts. These results show that the ex vivo evaluation of the sensitivity to induction drugs using primary blasts from patients exhibits a correlation with the response to induction chemotherapy in patients. These analyses would allow establishing a system to predict response to treatment and determine ex vivo susceptibility to new therapies under development, among which is phytotherapeutics.

## 1. Introduction

Acute leukemia (AL) is a neoplasm of hematopoietic origin that results from the uncontrolled clonal proliferation of abnormal myeloid or lymphoid progenitor cells arrested at different stages of differentiation and/or cells committed to the lymphoid, granulocytic, monocytic, erythroid, megakaryoblastic, and less frequently towards mastocytic, basophilic, and dendritic lineages [1, 2]. AL infiltrates the bone marrow and involves different tissues at the extramedullary level, which is associated with advanced disease and a worse prognosis. It produces progressive cytopenias and can occur de novo or be secondary to chemotherapy or myelodysplastic syndromes in the case of myeloid leukemias [3, 4].

Leukemic cell resistance to chemotherapy remains the leading cause of progression and death in patients [5, 6]. As a result of this, combinations of different drugs with different molecular targets have been used within therapeutic protocols. However, refractoriness to therapy, induction failure, and low survival are still observed. To select the best drug or combination of them to guarantee the patient response, in vitro tests have begun to be used to determine the sensitivity profiles to chemotherapy [7–11], and even of some natural extracts [12]. These sensitivity tests or platforms allow the selection of active chemotherapeutic agents to be used in patients and at the same time can facilitate the selection of potentially sensitive patients to participate in clinical studies of new molecules that are alternatives for the treatment of AL. Among these alternative therapeutics, herbal medicine stands out. It consists of the use of extracts or preparations derived from plants, some of which have shown an antileukemic effect both in vitro and in vivo, through different mechanisms [13–15].

Recently, our group has obtained and standardized two plant extracts (*Caesalpinia spinosa* and *Petiveria alliacea*) whose traditional use has been framed in the treatment of different types of cancer. The extract P2Et (obtained from *Caesalpinia spinosa*, or Dividivi) is enriched in polyphenols derived from gallic acid such as ethyl gallate and methyl gallate, among others [16]. P2Et has shown cytotoxic activity in different tumor cell lines, including leukemia lines, melanoma, and breast cancer resistant or sensitive to chemotherapy and antitumor activity in vivo in models of melanoma and breast cancer [17, 18]. The mechanisms involved in tumor death involve mitochondrial depolarization, activation of caspase-3, apoptosis dependent on intracellular Ca^++^, and consequent decrease in clonogenicity. Furthermore, when used in co-treatment with K562 cells with sub-lethal doxorubicin concentrations, P2Et increases sensitivity to the latter by a factor of four [16, 18–22].

The Anamu SC extract (obtained from *Petiveria alliacea*) presents some sulfur compounds (e.g., dibenzyl and tribenzyl sulfide) sterols, triterpenes, and flavonoids as its main compounds. *Petiveria alliacea* extract has shown cytotoxic activity in different tumor cell lines and in animal models of breast cancer. Among the mechanisms, the down- and upregulation of proteins involved in metabolism were observed, related to glycolysis and mitochondrial respiration decrease and the alteration of mitochondrial *β*-F1ATPase expression, and decrease in ATP and lactate production. Furthermore, it presents immunomodulatory activity on dendritic cells [23–27].

The ethnobotanical history of Anamu is more extensive than that of Dividivi regarding the treatment of leukemia. Anamu leaves and root powder have been used as an infusion by traditional medicine to treat rheumatism, spasmodic emesis, fever, and intestinal parasites [28]. Likewise, the infusion is used to treat leukemias and breast cancer, although the cytotoxic activity of the ethanolic extract against leukemic cells is not very high [29]. In contrast, there are a few studies of the Dividivi or other *Caesalpinia* species in terms of their activity in leukemias, although the *Caesalpinia sappan* species have been widely used in traditional Chinese medicine [30] and there is evidence of the mechanisms of action of some of its metabolites [31, 32].

Using this background, we wanted to assess whether the P2Et and Anamu SC extracts exhibited in vitro activity on primary leukemic cells treated alone or in conjunction with the chemotherapy of choice. Additionally, to evaluate the validity of our results, we analyzed whether the in vitro response to chemotherapeutics could be related to the induction response and the survival of the patients.

## 2. Materials and Methods

This study was approved by the Research and Ethics Committee of the Faculty of Medicine of the Hospital Universitario San Ignacio (FM-CIE-0206-16). All subjects gave written informed consent in accordance with the Declaration of Helsinki.

### 2.1. Patients

A total of 26 newly diagnosed patients (10 patients with acute myeloid leukemia (AML) and 16 patients with acute lymphoid leukemia (ALL)) and 6 with relapse, who attended the Hematology Service at the Hospital Universitario San Ignacio (HUSI) in Bogota, Colombia, between May 2016 and March 2018, were included in the study to assess the response of the leukemic cells to chemotherapeutics used in induction and to extracts. The patients were diagnosed and classified according to the hospital's clinical guidelines considering the immunophenotype results evaluated by flow cytometry, the morphological evaluation in the myelogram, the genetic alterations studied by conventional karyotype and/or molecular biology, and the clinical characteristics recorded in the histories. Eight patients presented an abnormal karyotype. 3 patients with B ALL were noted who had the translocation *t* (9; 22) (q34; q11) with BCR-ABL. 11 patients presented CNS infiltration, 10 ALL, and 1 AML. The chemotherapeutic protocol of choice for AML was 7 + 3 and for ALL the hyperCVAD. Idaflag was used in 3 patients, arsenic trioxide in 1, and azacitidine in 2 patients as palliative therapy in relapse patients. Peripheral blood or bone marrow samples were collected after signing the informed consent previously approved by the Institutional Review Board. The clinical characteristics of the patients are reported in Table 1.

Leukemic cells were obtained from bone marrow aspirate samples using Ficoll-Hypaque (Sigma Aldrich, USA). Viability was confirmed and should be greater than 95%. Subsequently, the cells were cultured in RPMI-1640 (Eurobio, Toulouse, France) supplemented with heat-inactivated fetal bovine serum (10%; Eurobio), 2 mM L-glutamine, 100 U/mL penicillin, 100 ug/mL streptomycin, 0.01 M Buffer Hepes, and 1 mM sodium pyruvate (Eurobio) and incubated in a humidified environment at 37°C and 5% CO_2_ until the test was done.

### 2.2. Cell Lines

The human leukemic cell line K562 (from a patient with chronic myeloid leukemia (CML)) was obtained from American type culture collection (Manassas, VA, USA). The human leukemic cells lines U937 (from a patient with monocytic differentiation-inducible histiocytic lymphoma) and Jurkat (from the peripheral blood of a 14-year-old boy with T-cell acute lymphoid leukemia) were provided by Dr. Hélène Moins‐Teisserenc (Laboratoire d'Hématologie Biologique, AP‐HP, Hôpital Saint Louis, 75010 Paris, France). Cells were grown in RPMI-1640 (Eurobio, Toulouse, France) supplemented with heat-inactivated fetal bovine serum (10%; Eurobio), 2 mM L-glutamine, 100 U/mL penicillin, 100 ug/mL streptomycin, 0.01 M Buffer Hepes, and 1 mM sodium pyruvate (Eurobio) and incubated in a humidified environment at 37°C and 5% CO_2_. Cell lines were proven mycoplasma-free using a MycoProbe Mycoplasma Detection Kit (R&D Systems) and maintained with ciprofloxacin (0.5 *μ*g/mL).

### 2.3. Reagents

The chemo drugs methotrexate (500 mg/20 mL : Pharmachemie), idarubicin (10 mg/5 mL : Pfizer), azacitidine (100 mg/5 mL : Tecnofarma), and arsenic trioxide (10 mg/2 mL : HB Human Bioscience) were obtained from the chemotherapy mixed center of the Hospital Universitario San Ignacio. The drugs were reconstituted in DMSO and stored at −80°C until use.

### 2.4. Plants Extraction and Characterization

The P2Et standardized extract was obtained as previously described [16], from the pods of *Caesalpinia spinosa* in LABFARVE laboratories, with quality of plant material according to FDA Guidelines for herbal medicinal products. The extract production is manufactured in a validated production process according to GMP. Quality of herbal drugs (starting material) is specified according to the relevant FDA Guidelines for herbal medicinal products [33]. This preparation is manufactured in a validated production process according to GMP. Comprehensive specifications and standardized production processes guarantee high batch-to-batch consistency.

Leaves of *Petiveria alliacea* were collected at Quipile, Cundinamarca, Colombia. The plant material was identified by Antonio Luis Mejia from the Colombian National Herbarium, voucher number COL 333406. The extract was obtained through supercritical fluids in La Corporación Universitaria Lasallista at 60°C, 400 bar, flow 30 Kg/h, and ethyl acetate 15% like cosolvent. The extract of Anamú (1.6 g), obtained by supercritical fluids extraction, was dissolved in a mixture of Hex : CH2Cl2 : MeOH (2 : 1:1 v/v/v). It was filtered and then separated by gel filtration chromatography on Sephadex LH-20 in a 2.5 × 60 cm column, using the same mixture as the solvent. Three fractions of 100 mL each were collected, FA (430 mg), FB (820 mg), and FC (320 mg). These were monitored by thin layer chromatography (TLC) on Sigel plates 0.25 mm, using Hex : AcOEt (9 : 1 v/v) and revealing them with vanillin/H_2_SO_4_ and subsequent heating.

The FB fraction was submitted to an in vitro biological assay (cytotoxicity in cell lines by MTT) showing a high activity and then was purified by chromatography in a column of 2 × 80 cms, using Sigel 60 for chromatography column, eluting with Hex : AcOEt 9 : 1 and collecting fractions of 100 mL each. In this way, six fractions were obtained, FB-1 to FB-6. The FB-1 fraction exhibited a strong garlic odor and was very volatile (which causes the rapid loss of material); the main compound was detected according to GC and 1H NMR. Because of the high volatility, and low polarity, a direct purification was carried out from a crude extract. Preparative TLC was carried out, using silica gel plates 0.5 mm thick and Hex : AcOEt 9.8 : 0.2 v/v as eluent, and two runs. A colored band of carotene and chlorophyll with Rf = 0.9 was noticed, and another blue band in UV with RF 0.6, and intense garlic odor, was also detected.

The stock solution of the extracts was prepared by reconstitution in 96.0% ethanol at a concentration of 25 mg/mL and stored at 4°C.

### 2.5. Analysis of Antioxidant Activity

The analyses were performed in AOX laboratories (aox lab.com).

#### 2.5.1. Oxygen Radical Absorbance Capacity (ORAC)

Sample preparation was carried out under the parameters established by AOAC 2012.23 [34]. Briefly, the extract, P2Et or Anamu SC, (0.5 g) was contacted with acetone and then diluted with PBS. Subsequently, 150 *µ*L of fluorescein and 25 *µ*L of the diluted extract were added to an ELISA plate, which was incubated for 30 minutes at 37°C. Then, 25 *µ*L of AAPH (2,2′-Azobis (2-amidinopropane) dihydrochloride) was added and the reading was performed on the spectrofluorimeter for 1 hour to obtain the emission (530 nm) and excitation (485 nm) data; then using the GEN5 software, the net AUC value was calculated. The antioxidant activity of the extracts is achieved with a calibration curve using the Trolox as standard.

#### 2.5.2. Ferric Reducing Antioxidant Power (FRAP)

FRAP was carried out according to Benzie et al. [35]. Aqueous solutions of known concentrations of Fe^+2^ (100–1000 mmol/liter) (FeSO_4_ ∗ 7H_2_O; Riedel de Haen) were used for the calibration. The FRAP solution was prepared with 10 mL of acetate buffer (300 mM) adjusted to *pH* 3.6. Subsequently, it was mixed with 1 mL of ferric chloride hexahydrate (20 mM) dissolved in distilled water and 1 mL of 2,4,6-Tris (2-pyridyl)-s-triazine (TPTZ) (10 mM) dissolved in HCl (40 mM). Subsequently, 10 *μ*L of the extract, 30 *μ*L of H_2_O, and 300 *μ*L of the FRAP solution were mixed and incubated at 37°C for one hour. Absorbance readings were made at 593 nm every 15 seconds during the monitoring period. The change in absorbance between the selected final reading and the reading was calculated for each sample and related to 593 nm of the Fe^+2^ standard solution tested in parallel. The activities of the samples under study are expressed as Fe^+2^ equivalents.

#### 2.5.3. ABTS Radical Scavenging Assay

The cationic radical ABTS•+ is obtained after the oxidation of ABTS (3-ethylbenz-thiazoline-6-sulfonic acid) (7 mM) with potassium persulfate (2.45 mM, final concentration) incubated at room temperature (±25°C) and in the dark for 16 hours. Once the ABTS•+ radical is formed, it is diluted with ethanol until an absorbance value within 0.700 ± 0.005 at 732 nm (wavelength of maximum absorption determined) is obtained. The extract is diluted with ethanol until an inhibition of 20 to 80% occurs when compared to the absorbance of the blank after adding 20 *µ*L of the sample. At 980 *µ*L of dilution of the ABTS•+ radical thus generated, the absorbance at 732 nm at 30°C is determined, 20 *µ*L of the sample is added, and the absorbance at 732 nm is measured again after 1 minute. Absorbance is measured continuously for 7 minutes. The reference synthetic antioxidant, Trolox, is tested at a concentration of 0–15 *µ*M (final concentration) in ethanol, under the same conditions. The results are expressed in TEAC (antioxidant activity equivalent to Trolox). This assay is also referred to as Trolox equivalent antioxidant capacity as it uses Trolox as a standard antioxidant.

### 2.6. Assessment of Cytotoxicity in Cell Lines by MTT

To evaluate the cytotoxic activity of P2Et and Anamu SC extracts, the MTT method, previously employed in our laboratory, was used [24]. Briefly, in a round-bottom 96-well microtiter plate, a cell density of 1 × 10^4^ cells per well/100 uL of supplemented RPMI 1640 medium was placed. The cytotoxic activity of each treatment was evaluated by performing 8 serial dilutions using concentrations of Anamu SC and P2Et extracts from 250 µg/ml. These treatments were incubated for 48 hours at 37°C, 5% CO_2_. Subsequently, two washes were performed and the MTT reagent (1 mg/ml) was added according to the manufacturer's recommendation. Finally, 100 *µ*l of DMSO was added and incubated for 20 min. Subsequently, the absorbance was read at 540 nm using a Multiskan^TM^ FC microplate photometer (Thermo Fischer Scientific, Waltham, MA, USA).

### 2.7. Ex Vivo Cytotoxicity Evaluation by XTT

The cytotoxic effect of the extracts (Anamu SC and P2Et) and the chemotherapeutic agents used (ALL induction: methotrexate; AML induction: idarubicin; Rescue ALL or AML : idarubicin, azacitidine, and arsenic trioxide) was evaluated using the XTT assay (2,3-bis (2-methoxy-4-nitro-5-sulfophenyl)-5-[(phenylamino) carbonyl]-2H-tetrazolium hydroxide) according to the manufacturer's instructions. 5 × 10^4^ primary leukemic cells were seeded per well in round-bottom 96-well plates in 100 uL of supplemented RPMI-1640 and were treated with chemotherapeutics or extracts at different concentrations for 24 h (P2Et/Anamu SC: 250 to 9.26 ug/mL, methotrexate/azacitidine: 100 to 3.70 uM and idarubicin/arsenic trioxide: 10 to 0.37 uM); subsequently, two washes with PBS were carried out at 1900 rpm, and the XTT reagent was added and incubated together with the cells for 8 h. Subsequently, the absorbance was read at 450 nm using a Multiskan^TM^ FC microplate photometer (Thermo Fischer Scientific, Waltham, MA, USA).

### 2.8. Quantification of the Response to the Drug and Statistical Analysis

Cell viability was calculated considering the absorbance values of the treatments, as well as that of the negative control as follows:

(1) $$\begin{array}{c} \text{\% viability}=\left(\frac{\text{absorbance value for each concentration of the treatment}}{\text{mean absorbance of negative control}}\right)\text{x}100\text{.} \end{array}$$

With the viability values, the IC50 was calculated by the methodology established by Chou [36]. For this, a logarithmic linearization was performed as shown below:

(2) $$\begin{array}{c} \text{log}\left(\frac{\text{\%}1-\text{viability}}{\text{\%viability}}\right)=\text{log}\left(\text{concentration}\right)\text{.} \end{array}$$

IC50 is the point of intersection of the line on the axis of concentration. In addition to the regression and according to the methodology established by Tyner [37], three situations that could occur to assign the IC50 value were considered; these are as follows: (i) the maximum concentration used for each treatment. The concentration does not decrease the viability by more than 50.0%; in this case, the IC50 value is reported as the highest concentration used for the treatment; (ii) the lowest concentration used for each treatment. The concentration reduces the viability by more than 50.0%; in this case, the IC50 value is reported as the lowest concentration used for the treatment; (iii) finding a good dose-response relationship. In this case, the maximum concentration of the treatment reduces the viability by more than 50.0% and at the minimum concentration of the treatment the viability is greater than 80.0%. The reported value corresponds to that calculated using the linear regression explained above. Finally, the curves were visually inspected to observe that the IC50 value corresponded to that observed in the cytotoxicity histogram.

### 2.9. Evaluation of the Synergistic or Antagonistic Activity of the Extracts

Leukemic cells (5 × 10^4^) cultured in supplemented RPMI were treated in the chemotherapeutic concentration range previously described and in each dilution of the chemotherapeutic agent, after P2Et or Anamu SC was added to a final extract concentration of 50 ug/mL and incubated for 24 hours at 37°C in 5% CO_2_. Subsequently, cell viability was evaluated by the XTT method.

### 2.10. Response Patterns of Treatments Evaluated by Heat Maps

IC50 values were normalized using *Z*-score and heat maps (R package gplots) were performed with the values obtained for each treatment. Comparisons were made between response patterns observed in the heat maps using the Mann-Whitney non-parametric *U* test to relate them to some clinical parameter of the patients.

## 3. Results

### 3.1. Extract Characterization

Among the identified compounds for P2Et are polyphenols derived from gallic acid, of which gallic acid, ethyl-gallate, and methyl-gallate are as previously reported [33]. UPLC-PDA chromatogram of P2Et is presented in Figure S1.

In this work, several compounds were isolated from an extract of Anamu leaves. In the first separation procedure using Sephadex LH-20, three fractions were obtained, FA (430 mg), FB (820 mg), and FC (320 mg). The FA fraction corresponds essentially to carotenes and chlorophyll, while the fraction FB exhibited an intense garlic odor and showed four purple/blue spots accordingly, TLC with Hex : AcOEt (9 : 1 v/v). Finally, the FC fraction corresponded to a pure compound that reveals brown color and whose structure by HPLC/MS and NMR indicates a C18-tri-unsaturated fatty acid.

Additional chromatographic separation of active fraction FB produced another six fractions. The FB-1 fraction with Rf 0.8 has an unpleasant smell and was very volatile; fractions FB-2 and FB-3 practically have no substances, while FB-4 and B-5 are the same compound (Rf 0.5, 16.6 mg), whose 1H NMR is indicative of a triterpene. Finally, the FB-6 fraction has two compounds (Rf 0.40, 22.5 mg) that were not possible to separate, which by the same previous technique allows detecting a sterol nucleus. The structures were assigned as described below. Due to volatility and low polarity, the main and odorous compound of the fraction FB-1 was purified from the crude extract by repeated preparative TLC in Hex : AcOEt 9.8 : 0.2. So, the Rf 0.60 band was scraped, extracted with methanol-chloroform, and evaporated to produce an unpleasant-smelling yellow oil; from 100 mg of crude extract, 5 mg of the compound was obtained by TLC. All HRMS spectra and 1H and 13C NMR signals agreed with the structure of dibenzyl disulfide, previously reported at the roots of that same plant [38]. The compound of the fractions FB4 and FB5 proved to be the triterpene isoarborinol. This substance is the main compound of the leaves of *P. alliacea* [39]. According to NMR spectra, the FB-6 fraction is a mixture of stigmasterol and sitosterol, according to MS and NMR spectra, and also was previously reported in this plant [40].

UPLC-PDA chromatogram of Anamu SC is presented in Figure S2. The compounds indicated in the chromatogram were identified as myricetin and dibenzyl disulfide (retention time of 14.0 min and 23.3 min, respectively). In addition to these compounds, 3*β*-isoarborinol, dibenzyl sulfide, stigmasterol, sitosterol, and octadeca-7,10,13-trienoic acid were also purified from this extract and identified using 1D and 2D NMR and APCI-Q-ToF-MS (data not shown).

### 3.2. P2Et Extract Is Highly Antioxidant unlike Anamu SC Extract

The antioxidant capacity of the extracts, measured by ORAC, FRAP, and TEAC methods, showed that the P2Et extract is highly antioxidant, unlike the extract of Anamu SC, which has a reduced antioxidant capacity (Table 2).

### 3.3. AL Patients Can Be Classified as Good or Bad Responders to Chemotherapy due to Their In Vitro Test Response Profile

**Figure 1:**
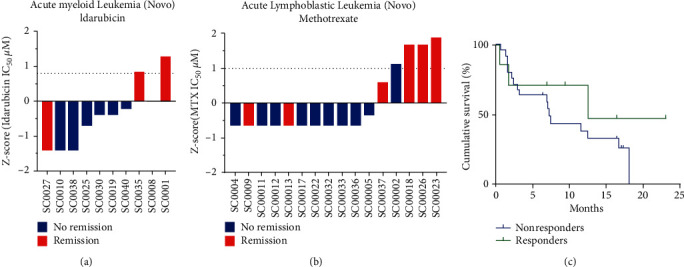
Relationship between the response to the ex vivo cytotoxicity test with the post-induction remission. (a) Response to idarubicin in primary cells of newly diagnosed acute myeloid leukemia patients. (b) Response to methotrexate in primary cells of newly diagnosed acute lymphoblastic leukemia patients. Red bars show patients who achieved post-induction remission and blue bars show patients who failed to achieve remission. The dotted line represents 1/5 of the IC_50_, where this cut-off point allows patients to be classified as good or bad responders. (c) Global survival, Kaplan–Meier survival curve of patients with acute leukemia according to the response observed in the ex vivo cytotoxicity test (*p*=0.295).

To make this classification between good responders (GR) and bad responders (BR), the IC50 of each of the drugs used in induction chemotherapy (idarubicin for AML and methotrexate for ALL) was calculated and IC50 values were normalized by *z*-score. For this, the mean and standard deviation of IC50 values were calculated and the difference between the IC50 value and the mean was divided by the standard deviation and multiplied by −1. According to the maximum concentration used for idarubicin (10 uM) and for methotrexate (100uM), those IC50s whose value is less than 1/5 of the maximum concentration used are considered GR, which in this case would be values less than 2 uM for idarubicin and less than 20 uM for methotrexate. If we take normalization into account, GR patients would be those with a z-score greater than 0.70 for idarubicin and 1.38 for methotrexate, and in contrast, the BR would be those with lower values than those indicated. Additionally, patients who achieved post-induction remission with red bars were identified as patients with AML (Figure 1(a)) or ALL (Figure 1(b)). For idarubicin-treated AML patients, we found that, of the 3 patients classified as in vitro as GR, 2 had responded correctly to induction therapy. For ALL patients, it was found that, of the 5 patients classified as GR, 4 of them had responded correctly to induction therapy.

The analysis of the response to induction chemotherapy was carried out according to the criteria defined by the European Leukemia Net (2017) [2], among which are blast count by myelogram less than 5%, absence of extramedullary disease, independence of transfusions, and blood count with normal counts. These results preliminarily suggest that the test could predict the response in vivo, but undoubtedly a larger sample should be evaluated to validate the results.

**Figure 2:**
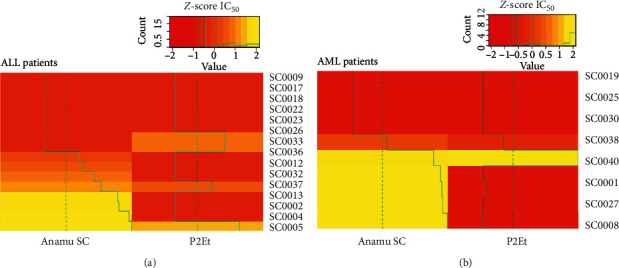
Resistance or sensitivity to treatment with extracts on cells of patients with acute leukemia. (a) Response of acute lymphoblastic leukemia (ALL) patients with the extracts and combinations. (b) Response of acute myeloid leukemia (AML) patients with the extracts and combinations. Red color indicates more resistance and yellow color more sensitivity. IC_50_ : inhibitory concentration 50.

Overall survival curves were made discriminating survival from in vitro response to drugs. A total of 25 BR patients and 7 GR patients were graphed. Survival curves were compared using the log-rank test. Patients classified as GR by the ex vivo test were found to have a median OS of 12.50 months versus 7.23 months for patients classified as poor responders. Even though no statistical significance was found (*p*=0.295) between the two groups, a greater overall survival rate (Figure 2) was observed in patients who responded positively to the ex vivo cytotoxicity test.

### 3.4. The Ex Vivo Response to Extracts and Chemotherapeutics Is Heterogeneous and Presents an Exclusive Pattern between Anamu SC and P2Et

The analysis of the cytotoxic activity of the Anamu SC and P2Et extracts showed that Jurkat cells were more sensitive to the treatment with the extracts compared to K562 and U937 cells. Furthermore, in the 3 lines, Anamu SC showed greater cytotoxic activity compared to P2Et (Table 3). The analysis of the activity of the P2Et and Anamu SC on the primary cells was performed with a heat map, plotting the IC50 of each treatment and normalized by *z*-score as indicated above. For comparison purposes, the map was organized starting with the responses to Anamu SC from those patients in whom no ex vivo response was observed to those where the response was optimal (IC50 less than 1/5 of the maximum concentration used). As seen in Figures 2(a) and 2(b), the primary cells of de novo AML and ALL patients show a clear segregation between those who respond or do not respond to Anamu SC and those who in contrast present a heterogeneous response for treatment to P2Et. Furthermore, it was shown that the primary cells of patients with ALL as AML are more sensitive to treatment with Anamu SC than with P2Et. Similarly, there is a group of patients who are resistant to both extracts.

### 3.5. Analysis of Ex Vivo Activity in ALL Patients

**Figure 3:**
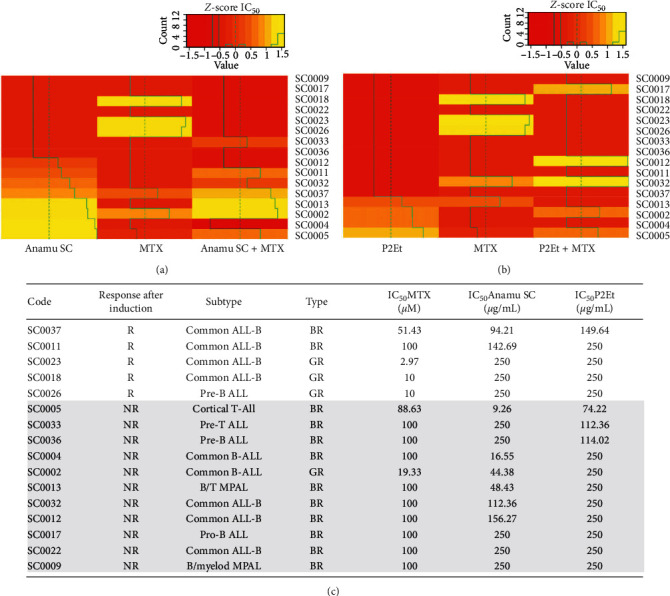
Resistance or sensitivity to treatment with extracts and methotrexate (MTX), individually or in combination, on cells of patients with acute lymphoblastic leukemia (ALL). (a) Response of ALL patients with Anamu SC, MTX, and their combination. (b) Response of ALL patients with P2Et, MTX, and their combination. Red color indicates more resistance and yellow color more sensitivity. (c) Response after chemotherapy induction of the ALL patients and the classification with the type of response in the in vitro test. IC_50_ : inhibitory concentration 50; R: response after induction chemotherapy; NR : non-response after induction chemotherapy; BR : bad responder; GR : good responder; MPAL : mixed phenotype acute leukemia.

When analyzing the effect of the combination of each of the extracts with conventional treatments for each leukemia, we can observe a pattern of specificity both for the type of leukemia, and with the joint activity between the extracts and the methotrexate. As seen in Figure 3, a better response to Anamu SC extract was observed in patients with ALL than to methotrexate and P2Et. Additionally, a group of patients (SC0018, SC0023, and SC0026) responded very well to methotrexate, but not to Anamu SC or P2Et. Interestingly, these three patients also showed antagonistic effects to the combinations between the drug and the extracts. Among the relevant biological characteristics, these three patients were found to have extramedullary infiltration at the central nervous system level. On the other hand, of the 4 patients who responded to P2Et ex vivo, 2 presented the BCR-ABL translocation and surprisingly, the other 2 are the only patients with a T-ALL phenotype that were included in the study. It was observed that in the combination with Anamu SC the response to chemotherapy improved in 6 patients and decreased markedly in 3. On the other hand, for the combination with P2Et, the response of chemotherapy improved in 5 patients and was lost in 3 patients.

A further analysis of the data, presented in the table contained in Figure 3, shows how (4/5) ALL patients who had a good response to induction (R) also showed a good response to ex vivo methotrexate (IC50 < 50 uM), corroborating what was shown in the survival curve. Interestingly, we observed that all the patients with a better ex vivo sensitivity to Anamu SC were found in the group of patients who did not respond to induction (IC50 < 50 ug/ml). If we consider that the test could predict the effectiveness of the therapy in patients, it would be thought that these patients who responded poorly to methotrexate both in the ex vivo test and in induction treatment could be candidates to receive the standardized Anamu SC extract, for which they presented a good response in the ex vivo test. Besides, the following arises: whether the lack of response to induction due to greater resistance to chemotherapy is accompanied by an increase in sensitivity to Anamu SC as a reflection of a change in tumor metabolic status. We will discuss this later.

### 3.6. Analysis of Ex Vivo Activity in Patients with AML

**Figure 4:**
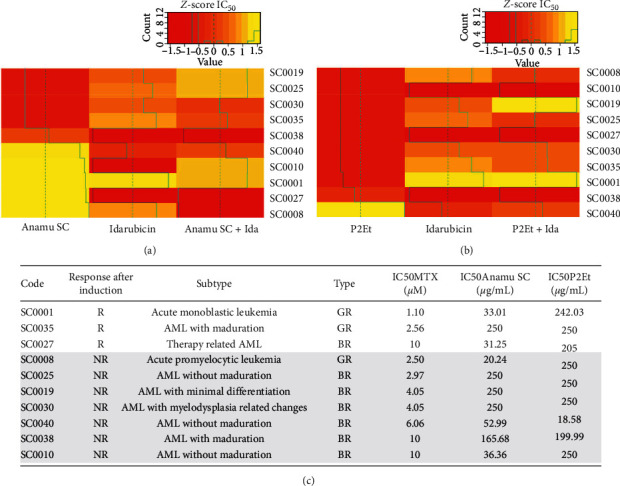
Resistance or sensitivity to treatment with extracts and idarubicin (Ida), individually or in combination, on cells of patients with acute myeloid leukemia (AML). (a) Response of AML patients with Anamu SC, Ida, and their combination. (b) Response of AML patients with P2Et, Ida, and their combination. Red color indicates more resistance and yellow color more sensitivity. (c) Response after chemotherapy induction of the AML patients and the classification with the type of response in the in vitro test. IC_50_ : inhibitory concentration 50; R: response after induction chemotherapy; NR : non-response after induction chemotherapy; BR : bad responder; GR : good responder.

In the primary cells of AML patients (Figure 4), the first thing that is observed is that a greater number of patients respond ex vivo to Anamu SC compared to P2Et. As for the patients with ALL, it is observed that a group of patients with AML that respond to Anamu do not present a response to idarubicin (SC0040, SC0010, and SC0027) and vice versa (SC0035, SC0030, SC0025, and SC0019); that is to say, a mirror ex vivo response seems to be presented. Contributing to this diversity of responses, it was interestingly observed that, in patient SC0010, when testing the combination of Anamu SC with idarubicin, the cytotoxic activity of Anamu is maintained, while in patient SC0008 Anamu SC seems to have an antagonistic effect, again showing that there are elements that determine the selectivity to one or the other treatment and that mixtures of these compounds without a previous study can lead to major therapeutic failures. For its part, the P2Et extract only showed activity in the cells of patient SC0040, which did not show significant chemotherapeutic sensitivity, and this sensitivity only partially improved in the presence of P2Et. Interestingly, we observed that the pattern of sensitivity or resistance to chemotherapy in conjunction with chemotherapy does not change substantially in the presence of P2Et, unlike that observed with Anamu SC.

Among the biological variables analyzed, no specific clinical characteristics related to differences in ex vivo responses were found, which could be associated with the low number of patients analyzed.

### 3.7. Ex Vivo Responses of Patients in Relapse

**Figure 5:**
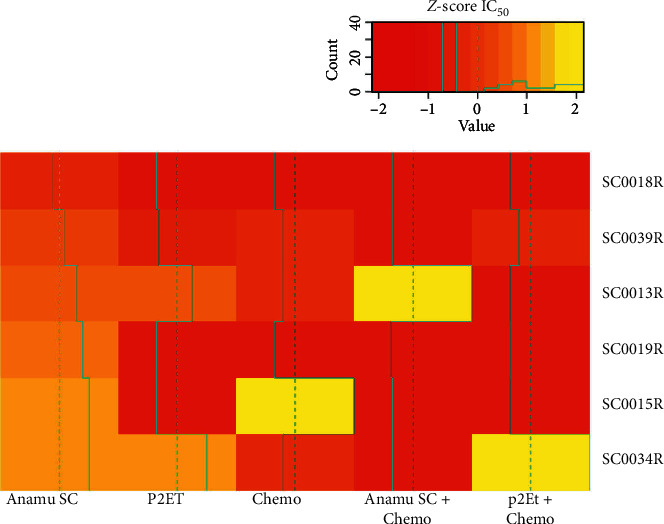
Response to extracts and chemotherapeutics in patients with relapse acute leukemia. Heat map showing resistance or sensitivity to treatment with extracts and chemotherapeutics individually or in combination on cells of patients with relapse acute leukemia. Acute lymphoblastic leukemia relapsed patients: SC0013 R, SC0015 R, SC0018 R (chemotherapy: idarubicin). Acute myeloid leukemia relapsed patients: SC0019 R, SC0034 R, SC0039 R (chemotherapy: azacitidine for SC0019 R and SC0039 R patients, and arsenic trioxide for SC0034 R patient). Red color indicates more resistance and yellow color more sensitivity. IC_50_ : inhibitory concentration 50.

Of all the patients analyzed, six of them were in relapse and were refractory to chemotherapy. In them, we observe that all are sensitive to ex vivo treatment with Anamu SC and only 2 respond to P2Et. Treatment with rescue chemotherapy (idarubicin, azacitidine, and arsenic trioxide) was only cytotoxic in patient SC0015 R (relapsed Pre-B ALL-B), who in turn is sensitive to Anamu SC but not to P2Et. In the combinations with the extracts, it is observed that the Anamu SC loses effectiveness in 5/6 patients except for the patient SC0013 R, in whom the combination is more favorable. In combination with P2Et, only patient SC0034 R substantially improves their sensitivity to chemotherapy (Figure 5). Although no biological pattern is observed to explain the differences, due in part to the low number of samples and the diversity in the leukemia phenotype as seen in Table 1, together with the lack of genetic analysis in all patients, these results show in an interesting way that patients refractory to chemotherapy are more sensitive to ex vivo treatment with Anamu SC extract, whatever their initial diagnosis is.

## 4. Discussion

Precision medicine seeks to define the correct treatment for each patient based on the biological characteristics of each one. Although this concept has been synonymous with individualized genomics, today there is important evidence showing that many different biological factors of genetic characteristics play a role in individual sensitivity and that these factors must be taken into account when analyzing the best therapy [41].

The ex vivo sensitivity study of leukemic blasts has recently been used by several research groups [7, 42, 43] allowing the discovery of new medications and the redirection of some personalized therapies. In this work, we show how a simple test that measures the metabolic activity of tumor blasts ex vivo can serve to apply the principle of precision medicine to the development of phytomedicines.

The test developed in our group allowed us to assess ex vivo sensitivity to induction chemotherapeutics used in the clinical protocols established in the guidelines for the treatment of ALL and AML. Furthermore, we were able to measure the ex vivo activity of two standardized plant extracts, which can serve as a basis for the development of phytomedicines for the treatment of AL. We were able to establish that there was a correlation of 70.5% between the patients classified as good responders according to the IC50 calculated for chemotherapeutics, and the remission to post-induction chemotherapy. We also observed that these patients had a greater overall survival than those in which no response to the cytotoxicity test was observed.

Regarding the activity of the extracts, we found that Anamu SC was more active than P2Et, on the primary cells of a significant proportion of patients with AML and ALL, both of primary diagnosis and in relapse. The Anamu has been extensively studied through time. Traditionally, there is talk of the “Managua case” where it was reported that a group of cows suffering from leukemia were put to pasture in places where Anamú grew abundantly and after a certain time they were cured [44]. Likewise, in 1981, Dr. Sergio Santana Sánchez in Cuba presented a detailed scientific study of 200 cases of evicted cancer and leukemia patients, who were treated with a mixture of different herbs, in which the Anamu was mainly found. The disappearance of the clinical and hematopoietic symptoms of the disease was reported for all cases, without observing any manifestation of intolerance or toxicity [45].

Based on the antecedents of antitumor use of Anamu, our group has been studying the mechanisms involved in this activity for the last 15 years. We have been able to confirm that the extract of *Petiveria alliacea* alters the cellular cytoskeleton as previously shown [46], further inducing cell cycle arrest in human melanoma cells, and cell death independently of the mitochondria. This activity was accompanied by a dysregulation of some proteins related to energy metabolism, cytoskeleton, and cell adhesion, analyzed through differential protein expression by HPLC-Chip/MS analysis [24]. These findings were subsequently confirmed in murine breast cancer 4T1 cells, where a decrease in glycolysis, mitochondrial respiration, and lactate and ATP production was also observed, with reduced expression of *β*-F1ATPasa [26, 27]. These and other activities of the Anamu have been recently revised [47], confirming the enormous potential of this plant in the treatment of cancer due to the intrinsic synergy of its components that allow it to act as a mitochondrial uncoupling and glycolytic inhibitor. These molecular targets have been implicated in the effective elimination of chemotherapy resistant primary AML cells which appear to be more sensitive to mitochondrial damage than other tumor blasts derived from solid tumors [48]. This is independent of the presence of leukemic stem cells [49] which may also have a rescue glycolytic metabolism [50]. The decrease in ATP production by both routes would lead to the death of the tumor cell due to energy deprivation.

In contrast to that observed for Anamu SC, we found a low cytotoxic activity of the P2Et extract against the primary blasts of AL, both lymphoid and myeloid. P2Et acts through different molecular mechanisms as previously described, which is evidenced by its very high antioxidant capacity in contrast to Anamu SC (Table 2). The decrease of intracellular ROS could then favor the selection of LSC (leukemic stem cells) ROS-low, which are more resistant to chemotherapy [51]. A recent study shows that standard chemotherapy increases ROS levels but that this is not enough to eliminate long-term LCS, which are the cause of relapse [52]. This is in line with the uncertainty about the use of antioxidants in conjunction with chemotherapy in the treatment of cancer [53]. Interestingly, with primary blasts, the best activity was against primary T-ALL cells as well as with the T-ALL tumor line (Jurkat) which was the most sensitive to the P2Et extract (IC50 : 49,50 ± 2,4 ug/mL). We had previously observed that P2Et had cytotoxic activity against HL-60 myeloid leukemic cells, and K562, although with low cytotoxic activity [16, 17]. It has been reported in the literature for T-ALL that decreased ROS leads to cell death [54] which would, in part, explain the activity of P2Et in these cells. On the other hand, the selective effect of P2Et on the primary cells of two patients with the BCR-ABL mutation was observed. Oxidative stress is known to regulate the phosphorylation of BCR-ABL contributing to its oncogenic activity, so that the decrease in ROS could decrease the activity of BCR-ABL inducing cell death [55].

The findings obtained with the extracts must be interpreted carefully if they are to be used for the benefit of patients. There are other factors such as pharmacokinetics and bioavailability [56], synergy or antagonism [57, 58], and the adverse effects that the consumption of natural products can have that have not been developed correctly [59] and that can affect the patient, reducing their quality of life and the effect of allopathic therapy of choice.

## 5. Conclusions

The ex vivo cytotoxicity of the chemotherapeutics used in induction on primary cells evaluated in the sensitivity platform is related to the remission and overall survival of patients with acute leukemia; additionally, different response profiles are observed for the Anamu SC and the P2Et in primary cells. The Anamu SC seems to have a greater response in AML and on the cells of those patients resistant to induction therapy and the response of the P2Et may be related to alterations of the oxidative balance at the cellular level in patients with ALL.

## Figures and Tables

**Table 1:** Clinical characteristics of patients with acute leukemia.

Novo acute leukemia									
Code	Sex	Age (years)	Type	Subtype	Genetic alteration	% Blast by flow cytometry	Karyotype	Extramedullary infiltration in CNS	Chemotherapy protocol
SC0001	M	38	AML	Acute monoblastic leukemia	—	25	Abnormal	Negative	7 + 3
SC0002	F	62	ALL	Common B-ALL	—	57.1	Normal	Negative	HyperCVAD
SC0004	F	56	ALL	Common B-ALL	t(9; 22) (q34.1; q11.2)	84.7	Abnormal	Positive	HyperCVAD
SC0005	M	20	ALL	Cortical T-ALL	—	95.8	Normal	Negative	HyperCVAD
SC0008	F	66	AML	Acute promyelocytic leukemia	t(8; 21) (q22; q22.1)	89.9	Abnormal	Negative	7 + 3
SC0009	F	58	MPAL	B/Myeloid MPAL	—	68.6	Normal	Negative	HyperCVAD
SC0010	M	66	AML	AML without maturation	Trisomy 8/NPM1 (-) FLT3 (-)	61.2	Abnormal	Negative	7 + 3
SC0011	M	55	ALL	Common ALL-B	—	96.3	Normal	Positive	HyperCVAD
SC0012	M	35	ALL	Common ALL-B	—	97.2	Normal	Negative	HyperCVAD
SC0013	M	32	MPAL	B/T MPAL	—	96.4	Normal	Positive	HyperCVAD
SC0017	M	52	ALL	Pro-B ALL	—	76	Normal	Negative	HyperCVAD
SC0018	F	20	ALL	Common ALL-B	—	84.6	Normal	Positive	HyperCVAD
SC0019	M	68	AML	AML with minimal differentiation	—	92.1	Normal	Negative	7 + 3
SC0022	F	19	ALL	Common ALL-B	—	73	Normal	Negative	HyperCVAD
SC0023	M	51	ALL	Common ALL-B	—	97.6	Normal	Positive	HyperCVAD
SC0025	M	69	AML	AML without maturation	NPM1 (-) FLT3 (-)	76.9	Normal	Negative	7 + 3
SC0026	F	19	ALL	Pre-B ALL	t(9; 22) (q34.1; q11.2) 84.1		Abnormal	Positive	HyperCVAD
SC0027	M	19	AML	Therapy-related AML	—	51	Normal	Negative	7 + 3
SC0030	F	69	AML	AML with myelodysplasia related changes	inv(17)	90.1	Abnormal	Negative	7 + 3
SC0032	F	23	ALL	Common ALL-B	Hyperdiploidy	91	Normal	Negative	HyperCVAD
SC0033	F	60	ALL	Pre-T ALL	—	87.9	Normal	Negative	HyperCVAD
SC0035	F	22	AML	AML with maturation	NPM1 (-) FLT3 (-)	46.6	Normal	Negative	7 + 3
SC0036	F	58	ALL	Pre-B ALL	t(9; 22) (q34.1; q11.2)	82.54	Abnormal	Positive	HyperCVAD
SC0037	M	55	ALL	Common ALL-B	t(9; 22) (q34.1; q11.2)	92.3	Abnormal	Positive	HyperCVAD
SC0038	M	73	AML	AML with maturation	—	62.66	Normal	Negative	7 + 3
SC0040	F	66	AML	AML without maturation	NPM1 (-) FLT3 (-)	77.6	Normal	Negative	7 + 3
*Relapse leukemia*									
SC0013 R	M	32	MPAL	B/T MPAL	—	—	Normal	Positive	Idaflag
SC0015 R	F	22	ALL	LLA-B Pre-B	—	93.2	Normal	Negative	Idaflag
SC0018 R	F	20	ALL	Common ALL-B	—	79.2	Normal	Positive	Idaflag
SC0019 R	M	68	AML	AML with minimal differentiation	—	95.1	Normal	Negative	Azacitidine
SC0034 R	M	37	AML	Acute promyelocytic leukemia	APL without PLM-RARa	78	Normal	Positive	Arsenic trioxide
SC0039 R	M	75	AML	AML with maturation	—	22	Normal	Negative	Azacitidine

F : female; M : male; AML : acute myeloid leukemia; ALL : acute lymphoblastic leukemia; MPAL : mixed phenotype acute leukemia; NPM1 : nucleophosmin 1; FLT3 : FMS-like tyrosine kinase 3; PLM-RARa : PML-retinoic acid receptor alpha.

**Table 2:** Antioxidant capacity of P2Et and Anamu SC extracts determined by ORAC, FRAP, and TEAC.

	ORAC (*μ*mol Trolox/100 g)	FRAP (mEq ascorbic acid/100 g)	TEAC (mEq Trolox/100 g)
P2Et	733539.59	55457.75	329036.77
Anamu SC	17455.51	258.53	612.96

**Table 3:** Cytotoxic activity of the extracts on the tumor lines: K562, U937, and Jurkat.

Cell lines (IC_50_)			
Treatments	K562	U937	Jurkat
Anamu SC (*μ*g/mL)	75.4 ± 9.3	96.14 ± 1.4	13.14 ± 4.1
P2Et (*μ*g/mL)	154.6 ± 21.4	102.3 ± 14.5	49.5 ± 2.4

## Data Availability

The data used to support the findings of this study are available from the corresponding author upon reasonable request.

## Abbreviations

**AL**: Acute leukemia
GR: Good responder
BR: Bad responder
AL: Acute leukemia
AML: Acute myeloid leukemia
ALL: Acute lymphoid leukemia
MPAL: Mixed phenotype acute leukemia
NPM1: Nucleophosmin 1
FLT3: FMS-like tyrosine kinase 3
PLM-RARa: PML-retinoic acid receptor alpha
ORAC: Oxygen radical absorbance capacity
FRAP: Ferric reducing antioxidant power
TEAC: Antioxidant activity equivalent to Trolox
LSC: Leukemic stem cell
MTX: Methotrexate
Ida: Idarubicin
IC_50_: Inhibitory concentration 50
R: Response after induction chemotherapy
NR: Non-response after induction chemotherapy
F: Female
M: Male.

## Supplementary Materials

Supplementary Materials Figure S1: UPLC-PDA chromatogram at 274 nm of P2Et extract. (a) Gallic acid, (b) methyl gallate, (c) ethyl gallate. Figure S2: UPLC-PDA chromatogram at 274 nm of Anamu SC. Peak identification: (a) myricetin, (b) dibenzyl disulfide.
