# Supplementary material for: Preferential Activity of Petiveria alliacea Extract on Primary Myeloid Leukemic Blast
Source: Evid Based Complement Alternat Med. 2020 Dec 11;2020:4736206. doi: 10.1155/2020/4736206 (PMC7787761; doi:10.1155/2020/4736206)
Supplement: Supplementary Materials — Figure S1: UPLC-PDA chromatogram at 274 nm of P2Et extract. (a) Gallic acid, (b) methyl gallate, (c) ethyl gallate. Figure S2: UPLC-PDA chromatogram at 274 nm of Anamu SC. Peak identification: (a) myricetin, (b) dibenzyl disulfide. [file 4736206.f1.zip › 4736206.f1/Figure S2.docx]

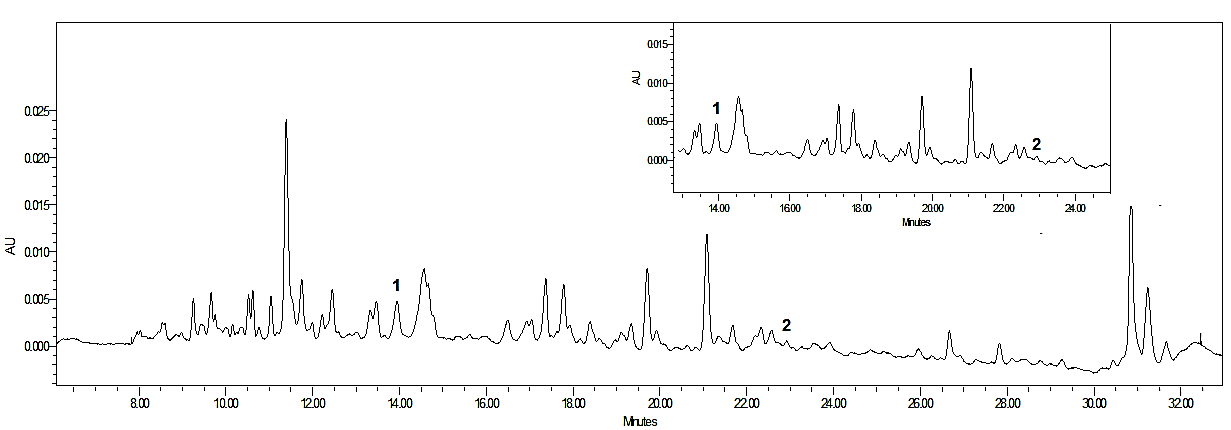


Figure S2. UPLC-PDA chromatogram at 274 nm of Anamu SC. Peak Identification: (1), myricetin; (2), dibenzyl disulfide.
